# Cross-Sectional Survey of the Amount of Sugar and Energy in Chocolate Confectionery Sold in the UK in 1992 and 2017

**DOI:** 10.3390/nu11081798

**Published:** 2019-08-03

**Authors:** Kawther M. Hashem, Feng J. He, Sarah A. Alderton, Graham A. MacGregor

**Affiliations:** Wolfson Institute of Preventive Medicine, Barts and The London School of Medicine & Dentistry, Queen Mary University of London, Charterhouse Square, London EC1M 6BQ, UK

**Keywords:** sugars, chocolate confectionery, reformulation

## Abstract

The study aimed to compare the sugar (1992, 2017) and energy (2017) content of chocolate confectionery available in the UK between 1992 and 2017 using cross-sectional surveys. All major UK retailers operating at the time were included. Sugar content in 1992 was obtained from a booklet and sugar and energy content from 2017 were collected from product packaging in-store. In 1992, the average sugar content of chocolate confectionery was 46.6 ± 10.3 g/100 g and in 2017 it was 47.3 ± 12.1 g/100 g. Sugar content ranged from 0.5 to 75.2 g/100g, with large variations between different categories of chocolate and within the same category of chocolate. There were 23 products found in both 1992 and 2017. The average sugar content per 100 g for these products was 44.6 ± 9.4 g in 1992 and 54.7 ± 6.3 g in 2017, representing a 23% increase in sugar content (*p* < 0.001). The results show that the sugar content of chocolate confectionery has increased since 1992, which is concerning. However, they also suggest sugar levels can be reduced because (a) lower sugar versions of the same products existed in 1992 and (b) there is a large variation in sugar and energy content between different categories of chocolate and within the same category in 2017.

## 1. Introduction

In July 2015, the Scientific Advisory Committee on Nutrition (SACN) in the UK recommended that average free sugars (sugar) intake, across the UK population, should not exceed 5% of total energy intake [1]. SACN’s advice was based on the need to reduce obesity, type 2 diabetes and dental caries risk [2,3,4,5,6,7,8,9,10].

In 2014, average intakes of sugar exceeded recommendations in all age groups [11]. The mean sugar intake in adults was 60 g per day, equivalent to 12% of daily energy intake. In children, the average sugar intake was 54 g (13%) per day in 4–10 year olds and 73 g (15%) per day in 11–18 year olds [11].

In order to reduce sugar intake (and therefore obesity and tooth decay) and help consumers to follow the principles of the Eatwell guide, the UK government published *Childhood Obesity: A Plan for Action (2016)*, in which a reformulation programme for sugar was included. The programme, led by Public Health England, asked manufacturers to reduce sugar by 20% by 2020 in each of the nine food and drink categories that contribute the most sugar in children’s diets, such as breakfast cereals, yoghurts, cakes, biscuits, morning goods, puddings, sweet spreads, sweet confectionery, ice cream and chocolate confectionery.

As part of the sugar reduction programme, companies can choose to achieve the 20% reduction by reformulating their products (without increasing overall calories), reducing portion size or promoting their lower sugar products [12]. Sales weighted averages (SWA) were calculated by weighing the sugar level of individual products against their volume sales; a high-selling product with high sugar levels drives the SWA upwards, whereas a high selling product with a low sugar level drives it downwards. SWA for chocolate confectionery is currently 54.6 g of sugar per 100 g, with the aim of bringing it down to 43.7 g by 2020 [12]. The SWA allows for flexibility in the sugar levels in different products within a category, e.g., a chocolate manufacturer can continue to sell a high sugar chocolate product if the remainder of their portfolio is lower. However, if the high sugar product is a big seller, the amount of sugar will have to be reduced through reformulation or reduced price promotions to reduce sales [12]. The calorie SWA was 200 kcal per 100 g and the cap for a single serve of chocolate was set at a maximum of 250 kcal [12].

This research aims to (a) compare sugar content in chocolate confectionery between 1992 and 2017, (b) evaluate the sugar and energy content of chocolate confectionery sold in the UK, (c) report the variability in sugar and energy content in 2017, (d) assess the sugar content in relation to the UK’s new daily recommendation for sugar intake and by chocolate manufacturers in the UK in 1992 and 2017 and (e) compare current serving sizes to the maximum calorie cap of 250 kcal suggested in the sugar reduction programme.

## 2. Materials and Methods

### 2.1. Data Collection

The sugar content from 1992 was obtained from a booklet published in 1992 by Octavo called *A–Z of Shopping; Guide to Good Health. What’s in your shopping basket?* The sugar and energy content from 2017 was collected from product packaging in one large outlet in London for each of the major UK supermarket chains (Aldi, Asda, Lidl, Marks and Spencer, Morrisons, Sainsbury’s, Tesco, The Co-operative and Waitrose). These supermarkets collectively hold over 93% of the grocery market share [13]. For each product in 2017, the data collected included the company name, product name, pack weight, portion/serving size, total sugars (g) and energy (kcal) per 100 g and per portion/serving. Data on total energy (kcal) content was collected to encompass the fat, carbohydrate, protein and sugar contents of products.

It was assumed that the ‘total sugars’ on the nutrition label of products represented the ‘free sugars’ content in chocolate confectionery. However, the definition of free sugars is new and not aligned with current claims and nutrition labelling on packaging. Free sugars includes all monosaccharides and disaccharides added to foods by the manufacturer, cook or consumer, plus sugars naturally present in honey, syrups and unsweetened fruit juices. It excludes lactose when naturally present in milk and milk products, as well as sugars contained within the cellular structure of foods (i.e., whole fruits and vegetables). Therefore, without the recipes of products, it is difficult to estimate the actual free sugars content of products that contain milk and/or dried fruit, but it was used as a proxy for free sugars anyway.

### 2.2. Product Categories

Products in 2017 were categorised by type (Table 1). The products were also categorised separately into the supermarkets’ own labels and brands and by manufacturer (if they had more than five products in both 1992 and 2017). Some products contributed to the manufacturer average sugar content and overall results but not to the category results, because they did not fit into a specific category and there were too few products to set a specific category for those products (Appendix A). Within ‘single serve’ products, duo products were included, since it was assumed that most consumers would consume both servings on one occasion (applied to one product). To check the accuracy of the data, each category was screened for outliers; the lowest and highest values in each category were checked with the original pack photos.

### 2.3. Inclusion/Exclusion Criteria

We included all chocolate except for selection/assortment boxes, seasonal products, such as Easter eggs, advent calendars and chocolate bunnies/snowmen/reindeer, and chocolate containing alcohol.

### 2.4. Analysis

*Per 100 g*: Some brands sell the same formulation in different pack sizes. The 100 g data used in the study only included one example, regardless of the different pack sizes. However, sometimes the product name was the same, which implied it was the same formulation/product, but the products seemed to have different nutritional contents per 100 g. In such cases, the products were different formulations and therefore considered as separate products.

*Per serving*: The per serving data included all the different per serving/chocolate/portion information available, or pack size (if one pack was equal to single serve).

*High, medium and low criteria for sugar content*: The sugar content was compared to the UK front-of-pack colour-coded labelling criteria for foods. Colour coding for total sugar was based on the following thresholds: Red/high >27 g/portion or >22.5 g/100 g, amber/medium >5.0 to ≤22.5 g/100 g and green/low ≤5.0 g/100 g [14].

*Maximum sugar intake*: The sugar content was also compared to the maximum daily recommendation for sugar intake for adults (30 g/day) and 7–10 year-old children (24 g/day) [14].

*Calorie cap*: The energy content per serving was compared to the maximum calorie cap of 250 kcal suggested in the sugar reduction programme.

*Manufacturers*: the sugar and energy contents were compared between supermarkets’ own labels and branded manufactures and by individual manufacturers to assess manufacturers’ current average levels.

### 2.5. Statistical Analysis

A comparison was made between supermarkets’ own labels and branded products for sugar. The Mann–Whitney U test was used when the sample size was small and the data were not normally distributed (i.e. for 1992) and an independent samples t-test was used when the sample size was large and the data were normally distributed (i.e. for 2017).

For the purpose of assessing sugar levels in the same products since 1992, only products with data available in both years were included in this analysis. A paired t-test was used to examine whether there was a significant change in the sugar content from 1992 to 2017.

Data were reported as means, standard deviations (SDs) and ranges, as indicated. Significance in all tests carried out was deemed significant as being *p* < 0.05. The data were analysed using IBM SPSS software, Version 25 (Armonk, NY, USA). 

## 3. Results

### 3.1. Sugar per 100 g in 1992

A total of 44 products were included in the per 100 g analysis. The average sugar content was 46.6 ± 10.3 g/100 g. Branded chocolate confectionery had a slightly higher sugar content compared with supermarkets’ own labels (47.8 g vs. 44.8 g, *p* = 0.173), but the difference was not statistically significant. All of the chocolate products would receive a ‘red’ (high) front-of-pack label for sugar.

Among the manufacturers with five or more chocolate products in this study, Nestle’s product range contained the highest average sugar content and Marks and Spencer’s contained the lowest (Table 2).

### 3.2. Nutrient Content in 2017

A total of 617 products met the inclusion criteria.

#### 3.2.1. Sugar and Energy per 100 g in 2017

**Figure 1:**
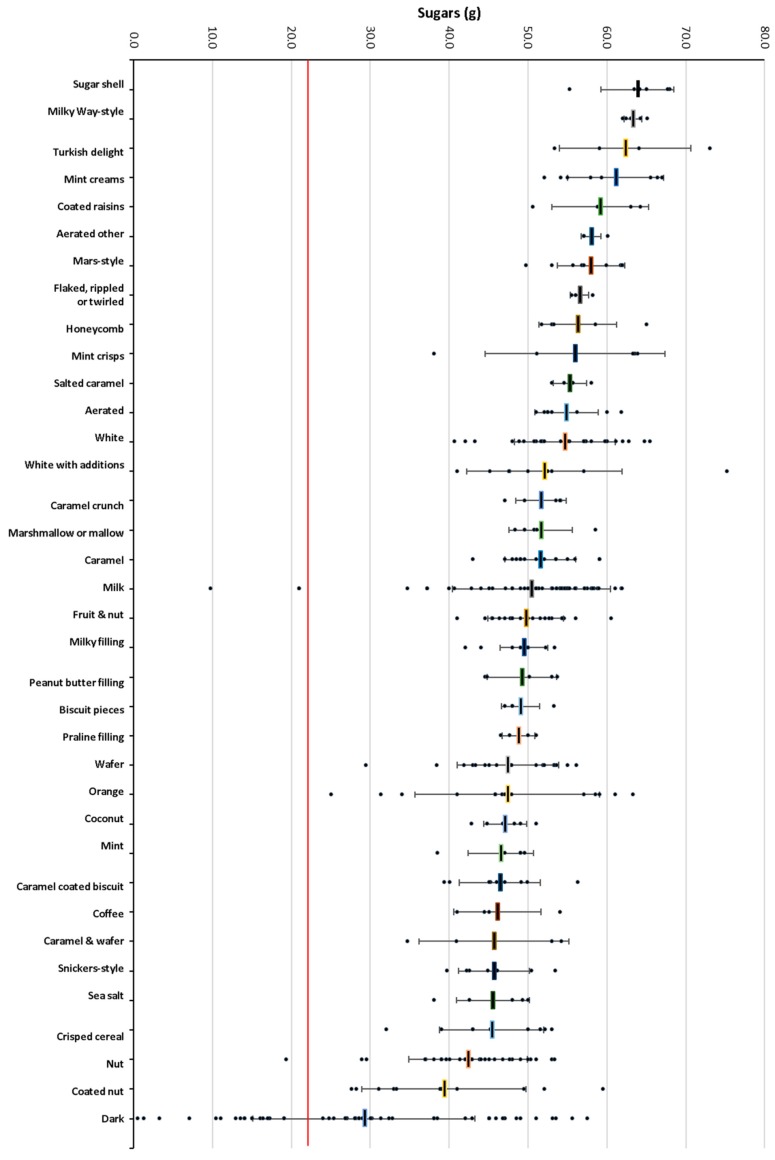
Sugar contents of different categories of chocolate confectionery in 2017 (g/100 g). The red line denotes red (high) criteria for sugar (>11.25 g). Values are individual products within each category (circle) with their means (rectangle) and standard errors represented by vertical bars.

A total of 527 products were included in the per 100 g analysis. Figure 1 and Table 3 show the sugar content in different categories of chocolate per 100 g. The average sugar content was 47.3 ± 12.1 g/100 g. There was a large variation in sugar content between different categories of chocolate and within the same category of chocolate, ranging from 0.5 to 75.2 g/100 g. Overall, 95% of chocolate products would receive a ‘red’ (high) front-of-pack label for sugar.

**Figure 2:**
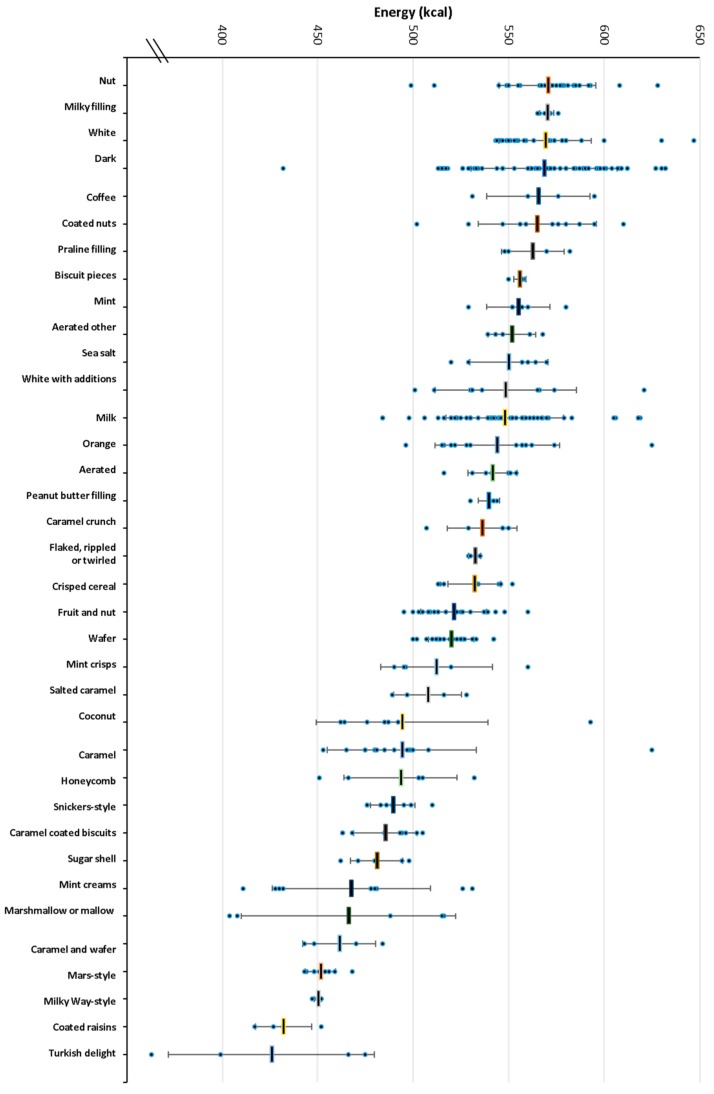
Energy contents of different categories of chocolate confectionery in 2017 (kcal/100 g). Values are individual products within each category (circle) with their means (rectangle) and standard errors represented by vertical bars.

Figure 2 and Table 3 show the energy contents per 100 g in different categories of chocolate. The average energy content in chocolate products was 533 ± 49 kcal/100 g. There was a large variation in energy content between different categories of chocolate and within the same category of chocolate, ranging from 121 to 647 kcal/100 g. On average, nut chocolate (570 ± 25 kcal/100 g) contained the highest amounts of energy and ranged from 499 to 628 kcal.

#### 3.2.2. Sugar and Energy per Serving in 2017

A total of 469 products provided nutritional information per serving/portion/chocolate. Serving size varied between different categories of chocolate confectionery and within the same category and ranged from 4.3 to 86 g. Among the single serve products, the serving size ranged from 14 to 55 g, while among the sharing bags format, the serving size ranged from 16 to 48 g. In the block chocolate format, the serving size ranged from 7 to 86 g.

The mean sugar content in chocolate confectionery was 12.9 ± 6.1 g/serving. Chocolate-coated Turkish Delight contained the highest sugar content per serving (25.2 ± 13.3 g), almost an adult’s entire maximum daily intake of sugar (Table 4).

On average, a serving of chocolate (15.6 ± 6.2 g sugar)—applied to single serve only—contained just over half (52%) of an adult’s (30 g/d) and over two thirds (65%) of a 7–10 year-old’s (24 g/d) maximum daily intake of sugar, with a range of 4.5 to 40.2 g

The mean energy content in chocolate was 142 ± 53 kcal/serving. Chocolate confectionery with peanut butter filling contained the highest calorie content per serving (222 ± 62 kcal) and mint crisps (62 ± 28 kcal) contained the lowest (Table 4).

Among the single serve chocolate confectionery, only four products exceeded the maximum calorie cap of 250 kcal per serving.

#### 3.2.3. Manufacturer

Branded chocolate confectionery had a slightly higher sugar content compared with supermarkets’ own labels, but the difference was not statistically significant (48.1 g vs. 46.5 g, *p* = 0.125).

Among the manufacturers with five or more chocolate products in this study (Table 5), Mars’ product range contained the highest average sugar content and Moo Free’s product range contained the highest energy content per 100 g.

### 3.3. Comparison of Changes in Sugar Content between 1992 and 2017

There were 23 products included in 1992 and 2017; the average sugar contents per 100 g were 44.6 ± 9.4 g and 54.7 ± 6.3 g (*p* < 0.001) respectively, which represents an increase of 23%.

## 4. Discussion

This study showed that the level of sugar in chocolate confectionery has increased since 1992, which is concerning from a public health nutrition perspective. Some may argue that the increase in sugar content was driven by consumer demand, but, as will be discussed later, marketing, advertisement and promotion of products also encourage demand for such products. Nonetheless, since products were lower in sugar in 1992, this may suggest that sugar can be reduced in chocolate confectionery. Without energy content data from 1992, it is difficult to be certain that these products were lower in energy as well as sugar compared to recent data.

Nevertheless, the large variations in sugar and energy content within the same category of chocolate in 2017 suggests that reformulation is possible. For instance, some manufacturers produce chocolates with far less sugar and calories than their competitors, as illustrated in Table 3 by the broad ranges within each category. This demonstrates that the amount of sugar and energy can be reduced through reformulation because similar, lower sugar content products are already available on the market. This research also makes available data on the sugar and energy content of chocolate confectionery in the UK in 2017 for future evaluation of the recently launched government-led sugar reduction programme.

In order to better understand how some manufacturers are able to produce chocolate confectionery products with lower levels of sugar and thus meet the aims of the sugar reduction programme, it is important to understand the function of sugar in chocolate and what sugar replacements are used. Sugar is added to chocolate to contribute sweetness, but also because it is a cheaper ingredient than cocoa or other types of fat. It is reported that a change in sugar content by just 1%–2% has large cost implications, which is why manufacturers may be reluctant to reformulate [15,16]. However, looking at the 2017 data, it appears there are a few products with extremely low sugar content (as little as 0.5%). Certainly, there have been studies to show that chocolate can be reformulated to reduce sugar and calories [17,18].There are some potential barriers in place for the food industry which can hinder, to a certain point, a gradual reduction in sugar, namely the European sweeteners directive (EC, 1994), which does not permit the use of sugar and some sugar replacements, such as polyols, in the same recipe mix. In this circumstance, a reduction in portion size may offer more scope as a means to reduce sugar (and calorie) consumption in this category, as suggested by Public Health England [12]. Nevertheless, pressure to review the sweeteners directive (EC, 1994) will aid chocolate manufacturers in creating products with gradual reductions in sugar and also provide more choice for consumers.

Sugar-free chocolates have recently become popular because of reduced calorific value and the fact that they are both non-cariogenic and suitable for diabetics [16,17]. The negative publicity surrounding sugar could also have played a role in the fact that 28% of people report they limit the amount of chocolate they eat due to the high sugar content [18]. There are tentative signs that manufacturers and retailers are placing a greater focus on this. Chocolate products with a low/no/reduced sugar claim grew in 2015 and 2016, with activity mainly from branded manufacturers [18]. Indeed, it was seen from the 2017 survey that there were a few chocolate confectionery products with extremely low concentrations of sugar, as low as 0.5%. However, these alternatives to well-known products, even after several years on the market, generally only account for a small proportion of sales and they are unlikely to change the market drastically [12].

In order to have a large impact, reformulation of existing products on the market is needed, owing to the huge volume of chocolate consumed; even small reductions would have a significant impact on sugar and energy intakes of the population. However, if the sugar reduction programme aimed to reduce childhood obesity, then perhaps the focus should be on the energy density of chocolate confectionery products and not just sugar content, because often the high-sugar products can be the lowest in energy content, as seen from the Nestle and Mars products in Table 5.

Furthermore, the 2017 survey showed that Mars, Nestle and Mondelez International are chocolate confectionery manufacturers with the highest sugar content on average. These are all multi-national companies, with large product portfolios. Some manufacturers may be reluctant to reformulate their products and reduce sugar and energy content due to fear of loss of sales. However, in recent years we have started to see many announcements of various reformulation efforts from such leading manufacturers [19].

In the future, a direct comparison could be made to track manufacturers’ reformulation performances over time. It also allows researchers, policymakers and the industry to identify which manufacturers offers products with the highest sugar or energy content on average and the products with the narrowest range in sugar, energy or both, which suggests that these manufacturers are providing limited choice to their customers in terms of sugar levels or energy-density in their products.

On the other hand, research shows that bigger and growing portion sizes result in more calories being consumed and it is estimated that if larger portions were removed from the diet completely, this could reduce energy intake by up to 16% [20]. Large serving sizes also distort the perception of what people view as a typical serving to consume [21,22]. Similarly, the recent increase in products sold in ‘sharing bag’ formats could encourage over-consumption of chocolate confectionery. The Grocer reported that one in four individuals do not share, but rather eat entire sharing bags themselves [23]. These can contain as much as 161 g sugar and 1142 kcal in a single bag. This seems counterproductive to previous efforts by the industry to standardise single serve confectionery to a 250 calorie cap back in 2014 [24]. The 2017 data shows that on average all categories were below the 250 kcal limit per serving. However, it is notable that, based on the ranges, the peanut butter filling and caramel-coated biscuit products are the two categories with products above this limit. In any case, while it is interesting to reflect on the energy content of single-serve products, these products are in decline, with ‘sharing bags’ and block chocolate now driving growth [25]. Since these formats are increasingly popular, manufacturers should look at packaging that encourages sharing or reduces the amount consumed in one go, e.g. resealable packs [26].

Furthermore, efforts may be hindered by the fact that the UK does not have standardised serving sizes for chocolate confectionery, unlike other countries such as Australia (set at 25 g) [27], the US and Canada (set at 40 g) [28,29]. As shown in this study, there is a lack of consistency among serving sizes of similar products, therefore manufacturer-recommended serving sizes are not always comparable across similar products and not always consistent with the portion size that consumers actually eat [30]. Displaying per serving and per pack information may confuse consumers and overcomplicate the information provided on packaging [30].

Therefore, consistent serving sizes that are aligned with dietary guidelines have the potential to gradually change norms to encourage healthier eating habits and could have a meaningful effect on population health [31]. Indeed, it may be timely that in Public Health England’s recent published analysis of the chocolate confectionery market, considered a single serve of chocolate confectionery as chocolate confectionery above 10 g or below 60 g [12]. To take this further, manufacturers should agree on a typical serving for similar formats, for example, a reasonable serving of chocolate confectionery in a sharing bag or a block of chocolate. Our data showed that not many products exceeded the maximum calorie cap per serve of 250 kcal [12]. This suggests the cap is not challenging enough and should be lowered.

In terms of sales, chocolate bars and blocks remain the top choices, each eaten by nearly two thirds of chocolate consumers [27]. Therefore, targeting those products with reformulation and portion size reduction may be the key in helping manufacturers reach their 20% reduction target. As seen from the 2017 data, there is a large variation in similar block chocolate products on the market per 100 g, such as white, dark and milk chocolate, which suggests there is scope to reduce sugar in these products, as well as energy. There is opportunity to reduce intake in these categories; 39% of chocolate consumers say a smaller portion/pack size would be a good alternative to a reduction in sugar [18]. However, transparency will be key to ensuring buy-in of such changes and to avoid consumer backlash, because 76% of consumers think chocolate brands should make it clear when they reduce the size/weight of the pack [18].

Aside from reformulation and portion size restrictions, evidence shows that consumption of chocolate is influenced by advertising and marketing [32]. Brands with high advertising spends see higher sales [33]. In 2016 there was an upsurge in advertising spend [18]. Advertising expenditure on chocolate confectionery increased by 15% in 2016, reaching £126 million in total [18]. Furthermore, many confectionery brands sponsor major sporting events, creating a unique marketing opportunity for them to boost sales. Cadbury, for instance, sponsored the London 2012 Olympics [34] and the Premier League in 2017/18 [34], whilst the England team sponsor was Mars during the UEFA (Union of European Football Associations) Euro 2016 tournament. Mars heavily promoted its range during and after the tournament in stores [35]. Therefore, introducing advertisement restrictions based on sugar or energy contents of products could also incentivise manufacturers to reformulate their products in order to advertise them.

Finally, another area that can have an impact on chocolate confectionery sugar content is front of pack nutrition labelling. This study showed that a high proportion of products would be labelled as red for sugars. If such a labelling system was enforced, some manufacturers may be incentivised to reformulate in order to avoid putting red labels on their products.

Increasingly, companies are calling for government-led regulations, since voluntary agreements are always led by progressive companies, often putting them at an economic disadvantage. Recently, Chairman and then-CEO of Nestle, Dame Fiona Kendrick, insisted the voluntary approach to tackling childhood obesity, as laid out by Public Health England, would not go far enough and called for government regulation to tackle the health crisis [26]. Various regulations, perhaps similar to the Soft Drinks Industry Levy, could be applied to encourage reformulation.

While there are many worthwhile findings in this study, it is important to acknowledge some of the limitations. The 1992 sugar content data used in this study was from a booklet published in 1992. We assumed the data included was a comprehensive representation of the products available on the UK market at the time. However, we are unable to verify this.

The 2017 data used were based on sugar and energy content data provided on product packaging labels in-store; hence, we relied on the accuracy of the data provided on the label. However, further studies could include sugar and energy contents determined through laboratory analysis to determine the accuracy of labels.

Furthermore, since we assumed the total sugars labelled on packaging was predominately free sugars, we may have overestimated the amount of free sugars in products that contained milk and/or dried fruit. Future analysis should seek to calculate the amount of milk and/or dried fruit used in a product to better understand the actual free sugars content of the product. This can be done by asking manufacturers to share their recipes or the amount of sugar and other ingredients used per 100 g.

This study did not analyse the fat, saturated fat, carbohydrate or protein contents of chocolate confectionery, but did collect and analyse total energy content in 2017, which would include the amount of energy coming from all these nutrients; therefore, any potential future reductions in the amount of energy can be from reductions in other nutrients too, as well as sugar.

Nevertheless, the results of this study are relevant and serve to document the sugar and energy content of chocolate confectionery sold in the UK, providing data to evaluate public health interventions, such as the Government’s sugar reduction programme, and act as an incentive for the chocolate confectionery industry to reformulate their products.

## 5. Conclusions

The results show that the sugar content of chocolate confectionery has increased since 1992, which is concerning from a public health perspective. However, the results also suggest that sugar levels can be reduced because (a) lower sugar versions of the same products existed in 1992 and (b) there is a large variation in sugar and energy content between different categories of chocolate and within the same category. This research also makes available data of the chocolate confectionery market in the UK for evaluation of the recently launched sugar reduction programme. A reduction in sugar and energy content and overall chocolate confectionery consumption could help reduce overall sugar and energy intake in the UK.

## Figures and Tables

**Table 1:** Descriptions and examples of chocolate confectionery categories.

Category	Description and Examples
Aerated	Products described as aerated milk chocolate, e.g., Cadbury Wispa and Aero
Biscuit pieces	Products described as dark or milk chocolate with biscuit pieces, e.g., Galaxy Cookie Crumble
Caramel	Products described as dark or milk chocolate with a soft caramel centre, e.g., Cadbury Dairy Milk Caramel
Caramel and wafer	Products described as a wafer layered with caramel covered in dark or milk chocolate, e.g., Nestle Drifters and Aldi Belmont Biscuits 8 Caramel Wafer Bars
Caramel coated biscuit	Products described as dark or milk chocolate with biscuit and caramel or toffee, e.g., Twix and Morrisons BicMix
Caramel crunch	Products described as dark or milk chocolate with crunchy caramel pieces and with or without salt, e.g., Tesco Milk Chocolate with Chunks of Caramel and Sea Salt
Coated nuts	Products described as dark or milk chocolate-covered nuts, e.g., Marks and Spencer Belgian Milk Chocolate Coated Peanuts
Coated raisins	Products described as dark or milk chocolate-covered raisins, e.g., Waitrose Chocolate Coated Raisins
Coconut	Products described as dark or milk chocolate with a coconut centre, e.g., Bounty
Coffee	Products described as dark or milk chocolate with coffee or coffee flavour, e.g., Heidi Dark Espresso
Crisped cereal	Products described as dark or milk chocolate-covered puffed rice/cereal, including flavoured chocolate, e.g., Nestle Crunch and Lidl Mister Choc Choco Thins Crispy Orange
Dark	Products described as plain dark chocolate, e.g., Morrisons Savers Dark Chocolate
Flaked, rippled or twirled	Products described as flaked, rippled or twirled milk chocolate, e.g., Galaxy Ripple and Cadbury Twirl Chocolate
Fruit and nut	Products described as dark or milk chocolate with fruit and nut, e.g., Lindt Excellence Orange Intense Dark with Almonds and Morrisons Dark Fruit & Nut Chocolate
Honeycomb	Products described as dark or milk chocolate-covered honeycomb, e.g., Crunchie
Marshmallow or mallow filling	Products described as dark or milk chocolate-coated marshmallow or mallow, e.g., Nestle Walnut Whip
Mars-style	Products described as milk chocolate with a soft nougat and caramel centre, e.g., Mars and Morrisons Apollo Bars
Milk	Products described as plain milk chocolate, e.g., Dairyfine Milk Chocolate Buttons
Milky filling	Products described as dark or milk chocolate-coated milky filling, e.g., Kinder Chocolate
Milky Way-style	Products described as whipped nougat covered in milk chocolate, e.g., Milky Way and Tesco Wild & Whippy
Mint	Products described as dark or milk chocolate with mint flavour, e.g., Green & Black’s Organic Dark Chocolate Mint
Mint creams	Products described as dark or milk chocolate with mint flavoured fondant filling, e.g., Elizabeth Shaw Dark Chocolate Mint Flutes
Mint crisps	Products described as mint flavoured dark or milk chocolate with sugar crunch/boiled sugar pieces, e.g., Elizabeth Shaw Dark Chocolate Mint Crisp
Nut	Products described as dark or milk chocolate with any type of nuts, e.g., Lindt Dark Hazelnut
Orange	Products described as orange-flavoured dark or milk chocolate, e.g., Tesco Plain Chocolate with a Hint of Zesty Orange
Aerated other	Products described as aerated chocolate non-milk flavoured, such as white chocolate, e.g., Nestle Aero Mint
Peanut butter filling	Products described as peanut butter covered with chocolate, e.g., Reese’s 3 Peanut Butter Cups
Praline filling	Products described as dark or milk chocolate with praline filling, e.g., Guylian Belgian Chocolate Original Praline
Salted caramel	Products described as dark or milk chocolate with a salted, soft caramel centre, e.g., Lindt Salted Caramel
Sea salt	Products described as dark or milk chocolate with sea salt, e.g., Green and Black’s Sea Salt Thin Dark Chocolate
Snickers-style/Nut Nougat Caramel	Products described as caramel and soft nougat with peanuts coated in milk chocolate, e.g., Snickers
Sugar shell	Products described as chocolate in a sugar shell, e.g., M & M’s Crispy and Smarties
Turkish delight	Products described as dark or milk chocolate with Turkish delight filling, e.g., Fry’s Turkish Delight
Wafer	Products described as chocolate bars with a wafer biscuit centre, e.g., KitKat
White	Products described as plain white chocolate, e.g., Waitrose Smooth Creamy Belgian White Chocolate
White with additions	Products described as white chocolate with additions, e.g., Hershey’s Cookies’n’Creme

**Table 2:** Sugar content in chocolate confectionery by manufacturer per 100 g in 1992.

Manufacturer	N	Sugars (g) Mean ± SD (Range)
Own label	18	44.8 (22.9–74.8)
Branded	26	47.8 (27.0–60.0)
Nestle	8	53.7 ± 3.0 (50.0–59.0)
Sainsbury’s	9	45.3 ± 14.2 (22.9–74.8)
Mondelez International	18	45.2 ± 9.9 (27.0–60.0)
Marks and Spencer	8	43.4 ± 9.7 (33.0–62.3)

**Table 3:** Sugar and energy contents of different categories of chocolate confectionery per 100 g in 2017.

Category	N	Sugars (g) Mean ± SD (Range)	Category	N	Energy (kcal) Mean ± SD (Range)
*Descending order*					
Sugar shell	6	63.9 ± 4.6 (55.2–67.9)	Nut	29	570 ± 25 (499–628)
Milky Way-style	6	63.3 ± 1.2 (62.0–65.1)	Milky filling	7	570 ± 4 (565–576)
Turkish Delight	4	62.3 ± 8.4 (53.3–73.0)	White	29	569 ± 24 (543–647)
Mint creams	10	61.1 ± 6.1 (52.0–67.0)	Dark	63	569 ± 38 (432–632)
Coated raisins	4	59.2 ± 6.2 (50.6–64.2)	Coffee	4	566 ± 27 (531–595)
Aerated other	5	58.0 ± 1.3 (57.0–60.1)	Coated nuts	11	565 ± 31 (502–610)
Mars-style	10	58.0 ± 4.3 (49.7–62.0)	Praline filling	4	563 ± 16 (548–582)
Flaked, rippled or twirled	4	56.6 ± 1.2 (55.5–58.2)	Biscuit pieces	5	556 ± 3 (550–558)
Honeycomb	6	56.3 ± 4.9 (51.7–65.0)	Mint	6	555 ± 16 (529–580)
Mint crisps	5	56.0 ± 11.4 (38.0–63.9)	Aerated other	5	552 ± 12 (539–568)
Salted caramel	4	55.3 ± 2.1 (53.0–58.0)	Sea salt	6	550 ± 20 (520–570)
Aerated	8	54.9 ± 4.0 (51.0–61.8)	White with additions	9	548 ± 37 (501–621)
White	29	54.7 ± 6.4 (40.6–65.4)	Milk	58	548 ± 31 (484–619)
White with additions	9	52.1 ± 9.9 (41.0–75.2)	Orange	14	544 ± 33 (496–625)
Caramel crunch	5	51.6 ± 3.2 (47.0–54.1)	Aerated	8	541 ± 13 (516–554)
Marshmallow or mallow	5	51.6 ± 4.0 (48.3–58.5)	Peanut butter filling	5	540 ± 6 (530–544)
Caramel	15	51.5 ± 4.5 (43.0–59.0)	Caramel crunch	5	536 ± 18 (507–550)
Milk	58	50.4 ± 10.0 (9.7–61.9)	Flaked, rippled or twirled	4	532 ± 3 (529–535)
Fruit and nut	22	49.7 ± 4.8 (41.0–60.5)	Crisped cereal	10	532 ± 14 (513–552)
Milky filling	7	49.5 ± 3.0 (44.0–53.3)	Fruit and nut	22	521 ± 17 (495–560)
Peanut butter filling	5	49.2 ± 4.4 (44.5–53.7)	Wafer	20	520 ± 12 (500–542)
Biscuit pieces	5	49.0 ± 2.4 (47.0–53.2)	Mint crisps	5	512 ± 29 (490–560)
Praline filling	4	48.8 ± 2.1 (46.5–51.0)	Salted caramel	4	508 ± 18 (489–528)
Wafer	20	47.4 ± 6.4 (29.4–56.1)	Coconut	7	494 ± 45 (462–593)
Orange	14	47.4 ± 11.7 (25.0–63.3)	Caramel	15	494 ± 39 (453–625)
Coconut	7	47.1 ± 2.7 (42.8–51.0)	Honeycomb	6	493 ± 30 (451–532)
Mint	6	46.6 ± 4.1 (38.5–49.5)	Snickers-style	8	489 ± 12 (476–510)
Caramel-coated biscuit	9	46.4 ± 5.2 (39.3–56.3)	Caramel-coated biscuit	9	485 ± 17 (463–505)
Coffee	4	46.1 ± 5.6 (41.0–54.0)	Sugar shell	6	481 ± 14 (462–498)
Caramel and wafer	4	45.7 ± 9.5 (34.7–54.2)	Mint creams	10	468 ± 41 (411–531)
Snickers-style	8	45.7 ± 4.5 (39.7–53.4)	Marshmallow or mallow	5	466 ± 56 (404–516)
Sea salt	6	45.6 ± 4.6 (38.0–50.0)	Caramel and wafer	4	461 ± 19 (443–484)
Crisped cereal	10	45.4 ± 6.6 (32.0–53.0)	Mars-style	10	452 ± 8 (443–468)
Nut	29	42.4 ± 7.5 (19.3–53.3)	Milky Way-style	6	450 ± 2 (447–452)
Coated nuts	11	39.3 ± 10.4 (27.6–59.5)	Coated raisins	4	432 ± 15 (417–452)
Dark	63	29.2 ± 14.1 (0.5–57.5)	Turkish Delight	4	426 ± 54 (363–475)
All products	527	47.3 ± 12.1 (0.5–75.2)	All products	527	533 ± 49 (121–647)

**Table 4:** Sugar and energy contents in different categories of chocolate confectionery per serving in 2017.

Category	N	Sugars (g) Mean ± SD (Range)	Category	N	Energy (kcal) Mean ± SD (Range)
*Descending order*					
Turkish Delight	4	25.2 ± 13.3 (13.3–40.2)	Peanut butter filling	4	222 ± 62 (135–277)
Sugar shell	6	20.8 ± 8.4 (9.0–29.4)	Snickers-style/nut nougat Caramel	8	204 ± 28 (171–250)
Coated raisins	4	19.6 ± 6.3 (12.7–25.2)	Caramel-coated biscuit	9	181 ± 63 (93–293)
Peanut butter filling	4	19.6 ± 5.1 (13.3–25.6)	Praline filling	3	172 ± 51 (114–210)
Snickers-style/nut nougat Caramel	8	19.0 ± 3.2 (15.1–24.7)	White with additions	7	164 ± 68 (88–233)
Honeycomb	6	17.9 ± 2.8 (14.6–21.5)	Turkish Delight	4	160 ± 51 (117–220)
Mars-style	10	17.5 ± 6.3 (10.0–25.6)	Sugar shell	6	159 ± 68 (66–232)
Caramel-coated biscuit	9	17.2 ± 5.8 (7.9–27.3)	Honeycomb	6	158 ± 33 (126–209)
Salted caramel	3	16.8 ± 4.3 (14.1–21.8)	Coated nuts	9	156 ± 42 (117–232)
Marshmallow or mallow filling	5	16.1 ± 1.9 (13.9–18.4)	Caramel crunch	3	154 ± 58 (106–219)
Flaked, rippled or twirled	4	15.8 ± 3.3 (12.0–19.2)	Salted caramel	3	153 ± 50 (122–211)
White with additions	7	15.6 ± 6.7 (8.3–26.3)	Nut	24	152 ± 57 (53–243)
Caramel	14	15.2 ± 4.5 (9.4–24.1)	Caramel and cereal	3	150 ± 42 (124–198)
Caramel crunch	3	14.9 ± 5.7 (10.8–21.4)	Flaked, rippled or twirled	4	148 ± 29 (115–175)
Praline filling	3	14.8 ± 4.7 (9.3–17.9)	Milk	53	147 ± 58 (30–282)
Coconut	6	14.7 ± 5.4 (10.7–25.5)	Fruit and nut	20	147 ± 42 (84–249)
Caramel and cereal	3	14.5 ± 4.1 (11.7–19.1)	Coconut	6	147 ± 42 (119–231)
Fruit and nut	20	14.2 ± 4.5 (9.1–26.0)	Marshmallow or mallow filling	5	145 ± 18 (127–171)
Mint creams	8	14.1 ± 8.2 (2.5–32.1)	Coated raisins	4	142 ± 38 (107–181)
Aerated	8	13.9 ± 4.5 (5.1–18.7)	Caramel	14	141 ± 44 (95–244)
Milk	53	13.7 ± 6.0 (1.9–29.4)	Wafer	20	140 ± 51 (87–228)
White	25	13.6 ± 4.7 (8.1–22.9)	White	25	140 ± 43 (82–229)
Milky Way-style	6	12.7 ± 1.9 (9.9–14.1)	Aerated	8	137 ± 45 (53–199)
Wafer	20	12.7 ± 4.8 (8.0–24.1)	Mars-style	10	136 ± 48 (80–205)
Orange	11	12.1 ± 5.1 (6.3–21.4)	Crisped cereal	10	136 ± 32 (103–221)
Aerated other	5	11.8 ± 3.9 (5.7–14.5)	Orange	11	132 ± 36 (83–195)
Crisped cereal	10	11.6 ± 3.6 (8.0–21.2)	Mint	5	123 ± 17 (110–145)
Nut	24	11.6 ± 5.0 (2.5–21.2)	Dark	54	123 ± 43 (31–251)
Coated nuts	9	10.7 ± 4.4 (6.9–21.0)	Sea salt	5	113 ± 26 (71–139)
Caramel and wafer	4	10.7 ± 3.4 (6.5–14.8)	Aerated other	5	112 ± 38 (56–142)
Mint	5	10.2 ± 1.9 (7.7–12.3)	Biscuit pieces	5	112 ± 45 (83–186)
Biscuit pieces	5	9.9 ± 3.8 (7.2–15.7)	Caramel and wafer	4	108 ± 30 (71–134)
Sea salt	5	9.3 ± 2.6 (5.3–12.5)	Mint creams	8	103 ± 54 (23–211)
Milky filling	7	8.2 ± 2.5 (3.0–11.2)	Milky filling	7	95 ± 28 (33–119)
Mint crisps	3	7.9 ± 3.5 (4.0–10.6)	Coffee	2	93 ± 31 (71–115)
Coffee	2	7.2 ± 2.6 (5.3–9.0)	Milky Way-style	6	90 ± 13 (72–99)
Dark	54	6.7 ± 4.4 (0.1–19.4)	Mint crisps	3	62 ± 28 (31–83)
All products	469	12.9 ± 6.1 (0.1–40.2)	All products	469	142 ± 53 (5–494)

**Table 5:** Sugar and energy contents of chocolate confectionery by manufacturer per 100 g in 2017.

Manufacturer	N	Sugars (g) Mean ± SD (Range)	Manufacturer	N	Energy (kcal) Mean ± SD (Range)
Own label *	284	46.5 ± 11.9 (1.2–75.2)	Own label *	284	539 ± 45 (399–632)
Branded **	243	48.1 ± 12.2 (0.5–67.9)	Branded **	243	526 ± 52 (121–647)
*Descending order*					
Mars	29	55.3 ± 6.0 (44.8–67.9)	Moo Free	5	579 ± 7 (568–585)
Nestle	42	54.8 ± 7.3 (29.4–66.4)	Montezuma’s Chocolates	8	576 ± 15 (547–601)
Mondelez International	55	54.3 ± 5.5 (45.0–65.5)	Lindt and Sprungli	20	569 ± 40 (507–647)
Aldi	54	50.3 ± 9.0 (14.0–65.0)	Ferrero	5	568 ± 9 (552–576)
Lidl	48	48.5 ± 11.2 (17.0–75.2)	Green and Black’s	13	566 ± 27 (510–630)
Ferrero	5	48.5 ± 5.6 (41.2–53.3)	Chocolate and Love	5	565 ± 19 (547–598)
Hershey Company	7	48.1 ± 4.8 (41.8–54.4)	Doisy & Dam	5	560 ± 10 (547–570)
ASDA	24	46.5 ± 10.5 (16.0–60.0)	Moser Roth	10	558 ± 28 (527–621)
Waitrose	16	45.5 ± 12.2 (27.6–67.0)	Marks and Spencer	50	553 ± 50 (399–632)
Morrisons	25	45.4 ± 10.1 (16.4–63.1)	Heidi Chocolat Suisse	6	551 ± 35 (515–600)
Marks and Spencer	50	45.2 ± 11.4 (16.0–73.0)	Waitrose	16	551 ± 60 (411–628)
Tesco	29	44.8 ± 13.2 (12.9–67.0)	ASDA	24	546 ± 35 (454–632)
Sainsbury’s	37	43.1 ± 16.4 (1.2–67.0)	Morrisons	25	541 ± 46 (451–619)
Heidi Chocolat Suisse	6	42.3 ± 18.5 (14.0–57.0)	Tesco	29	538 ± 37 (452–604)
Lindt and Sprungli	20	41.9 ± 12.9 (7.0–57.0)	Hershey Company	7	537 ± 13 (511–547)
Moser Roth	10	40.3 ± 10.2 (26.0–51.0)	Lidl	48	536 ± 48 (427–630)
Green and Black’s	13	39.2 ± 10.6 (13.5–51.0)	Sainsbury’s	37	533 ± 41 (444–596)
Moo Free	5	35.9 ± 3.3 (33.7–41.0)	Aldi	54	528 ± 41 (417–608)
Chocolate and Love	5	35.8 ± 10.5 (19.0–47.0)	Nestle	42	515 ± 26 (432–559)
Doisy and Dam	5	35.2 ± 13.4 (23.7–52.0)	Mondelez International	55	510 ± 41 (363–560)
Montezuma’s Chocolates	8	34.6 ± 17.4 (0.5–55.0)	Mars	29	499 ± 32 (443–559)

* Supermarket brand; store brand. ** Commercial brand.

## Appendix A

The following products contributed to the company averages and overall results but not category results, because they did not fit into a specific category.

Mars Revels 112 g

M and M’s Peanut 250 g

Nestle Caramac 4 bars

M and M’s Pretzel Chocolate Candies 32.3 g

Cadbury Dairy Milk Mixed Buttons 115 g

Nestle Caramac Giant Buttons 110 g

M and S Milk Chocolate Covered Pretzels & Popping Candy Popcorn 120 g

Nestle Pick and Mix 107 g

Marks and Spencer Milk Chocolate Coated Pretzels 60 g

Mars Revels 101 g

Mars Revels 173 g

M and M’s Peanut 140 g

Revels Treat Bag 78 g

Nestle Pick and Mix 107 g
